# Real-world first-line treatment and overall survival in non-small cell lung cancer without known EGFR mutations or ALK rearrangements in US community oncology setting

**DOI:** 10.1371/journal.pone.0178420

**Published:** 2017-06-23

**Authors:** Amy P. Abernethy, Ashwini Arunachalam, Thomas Burke, Caroline McKay, Xiting Cao, Rachael Sorg, David P. Carbone

**Affiliations:** 1 Flatiron Health, Inc., New York, New York, United States of America; 2 Duke University School of Medicine, Durham, North Carolina, United States of America; 3 Center for Observational & Real World Evidence (CORE), Merck & Co., Inc., Kenilworth, New Jersey, United States of America; 4 James Thoracic Oncology Center, Medical Oncology, The Ohio State University, Columbus, Ohio, United States of America; Universidade do Algarve Departamento de Ciencias Biomedicas e Medicina, PORTUGAL

## Abstract

**Purpose:**

To establish a baseline for care and overall survival (OS) based upon contemporary first-line treatments prescribed in the era before the introduction of immune checkpoint inhibitors, for people with metastatic non-small cell lung cancer (NSCLC) without common actionable mutations.

**Methods:**

Using a nationally representative electronic health record data from the Flatiron dataset which included 162 practices from different regions in US, we identified patients (≥18 years old) newly diagnosed with stage IV NSCLC initiating first-line anticancer therapy (November 2012- January 2015, with follow-up through July 2015). Patients with documented epidermal growth factor receptor (*EGFR*) or anaplastic lymphoma kinase (*ALK*) translocation were excluded. Anti-cancer drug therapy and overall survival were described overall, and by histology.

**Results:**

A total of 2,014 patients with stage IV NSCLC without known *EGFR* or *ALK* genomic tumor aberrations initiated systemic anticancer therapy, 22% with squamous and 78% with nonsquamous histology. Their mean (SD) age was 67 (10) years, 55% were male, and 87% had a smoking history. In nonsquamous NSCLC, carboplatin plus pemetrexed either without (25.7%) or with bevacizumab (16%) were the most common regimens; 26.6% of nonsquamous patients receiving induction therapy also received continuation maintenance therapy. In squamous NSCLC, carboplatin plus paclitaxel (37.6%) or nab-paclitaxel (21.1%) were the most commonly used regimens. Overall median OS was 9.7 months (95% CI: 9.1, 10.3), 8.5 months (95% CI: 7.4, 10.0) for squamous, and 10.0 months (95% CI: 9.4, 10.8) for nonsquamous NSCLC.

**Conclusion:**

The results provide context for evaluating the effect of shifting treatment patterns of NSCLC treatments on patient outcomes, and for community oncology benchmarking initiatives.

## Introduction

Lung cancer is the leading cause of cancer-related deaths in the United States (US) and worldwide: 221,200 new cases and 158,000 deaths from lung cancer were projected for 2015 in the US alone [1]. Non-small cell lung cancer (NSCLC) constitutes about 85% of all lung cancers and includes squamous cell carcinoma (~25–30%), nonsquamous carcinoma (adenocarcinoma, large cell, and undifferentiated carcinoma; ~70–75%), and non-small cell carcinoma, not otherwise specified (NSCC NOS; <5%) [1,2]. For approximately 80% of patients, the initial diagnosis of lung and bronchus cancer occurs when the cancer has already spread to regional lymph nodes (22%) or has metastasized (57%) [3]; the 5-year survival rates of patients with NSCLC and distant metastases (stage IV) are <5% [1–3].

The recommendations for systemic anticancer therapy for stage IV NSCLC vary according to tumor histology, the patient’s performance status, and driver oncogene biomarker status, most frequently epidermal growth factor receptor (*EGFR*) mutation and anaplastic lymphoma kinase (*ALK*) translocation [4–7]. Patients deemed candidates for systemic therapy who have stage IV NSCLC without an activating *EGFR* mutation or *ALK* translocation, representing approximately 85% of cases; guidelines recommend first-line (induction) therapy with a platinum-based doublet chemotherapy regimen using a carboplatin or cisplatin doublet. For nonsquamous NSCLC only, bevacizumab combinations are also acceptable [4,5,8]. Maintenance therapy is administered selectively for patients with stable disease or who respond to first-line chemotherapy after 4 cycles, per American Society of Clinical Oncology (ASCO) guidelines [9], or after 4–6 cycles, as recommended by NCCN guidelines [4]. One or more of the agents used in first-line induction therapy can be continued (continuation maintenance), or patients can be switched to a new agent (switch maintenance). The NCCN guidelines recommend bevacizumab and pemetrexed (alone or in combination) or gemcitabine for continuation maintenance and pemetrexed or erlotinib for switch maintenance. [4,5].

Current treatment patterns and their associated overall survival estimates provides context for interpreting clinical trial results and for clinical practice benchmarking initiatives. However, there are very limited data to describe treatment patterns and survival for metastatic NSCLC, which aimed to identify and exclude patients with actionable mutations using real world data sources. The most recent publications examined data through early 2010 [10,11], before the publication of continuation and switch maintenance trials [12–14]. The aim of this study was to describe overall survival (OS) with current first-line treatment for patients presenting with stage IV NSCLC without known *EGFR* or *ALK* tumor aberrations across a broad range of community oncology practices in the United States (US). The time period of the study is focused on patients diagnosed between November 1, 2012, to January 31, 2015, to set a baseline for care and OS based upon contemporary first-line treatments prescribed in the era before immune checkpoint inhibitors became prevalent in the US.

## Methods

### Data source

This was a retrospective observational study of electronic health record (EHR) data from the Flatiron Health database [15]. The EHR data, refreshed on a monthly basis, are anonymized for research use and include structured data (e.g., cancer-related diagnoses and staging, laboratory data, medications) and abstracted data derived from unstructured documents residing in the EHR (e.g., physicians’ notes, radiology/pathology/biomarker reports, discharge summaries). At the time of this study, the overarching Flatiron Health database represented approximately 220 cancer practices and practice groups in the overall network predominantly representing the community oncology clinical setting the dataset represented approximately 700 different sites of care, 1,700 clinicians distributed nationally, and managing 750,000 patients with active cancer. In the study dataset, there were 162 unique practices. The geographic distributions of the practices were 37.7% from Southern region, 23.5% and 21.9% from Northeast and Midwest, and 15.4% were from Western region of the country. Practices are defined by a single tax identifier. Group practices with multiple locations but a single tax identifier are represented as one practice. Flatiron uses a technology-enabled abstraction and multi-pronged quality assurance approaches to generate research-ready datasets [16, 17]. The combination of a nationally representative population plus structured and unstructured data processing leads to a nationally generalizable sample of NSCLC patients in terms of age, gender, and geographic location [16].

Central Institutional Review Board approval of the study protocol was obtained prior to study conduct. Informed consent was not required or possible as this was not an interventional study, and the anonymized data in the Flatiron EHR database are well-protected against the release of personal information in accordance with the Health Information Portability and Accountability Act [18].

### Patients and study design

Eligible patients for this study were adults (≥18 years) with a new diagnosis of NSCLC presenting with stage IV disease from November 1, 2012, to January 31, 2015, with two documented clinical visits on or after January 1, 2013 and with subsequent initiation of anticancer therapy; eligible patients were also treatment naïve, with no record of anticancer therapy during the prior 6 months. The population was intentionally limited to those people who were metastatic (stage IV) at diagnosis in order to reduce any risk of survival bias that may be encountered when recurrent metastatic patients are incorporated in an EHR database study. The initiation of anticancer therapy was defined as the *index date*, and patient data were followed until July 31, 2015, thereby providing minimum 6 months’ of follow-up for each patient. In addition, eligible patients had *EGFR* wild-type disease, negative *ALK* translocation status, or unknown *EGFR* mutation and *ALK* translocation status (defined as results pending or unknown, or an unsuccessful/indeterminate test). Patients with a known *EGFR* mutation and/or *ALK* translocation and those with NSCC NOS histology were excluded. Patient data were followed until death, loss to follow-up, or the end of the study period (July 31, 2015).

### Study endpoints

Patient demographic and clinical characteristics and their first-line induction and, when available, first-line continuation maintenance therapies for stage IV NSCLC were obtained from the Flatiron database. Patient characteristics included sex, birth year (used to derive age), race, smoking status, height and weight at the index date (used to derive body mass index [BMI]), and US census region. Disease-related variables included date of diagnosis, NSCLC stage at diagnosis, histology, and *EGFR* and *ALK* biomarker testing status at the index date.

Information about anticancer therapy included the dates and prescribed regimens, including drug names, route, dosage, and units. This information was used to derive a variable defining treatment setting (namely, line of therapy) according to predefined rules for induction and continuation maintenance regimens, described in detail in the online supplement. Treatment duration was defined as the time from regimen initiation to the last dose of drugs within the regimen and was summarized for patients receiving only first-line induction therapy as well as those receiving first-line induction followed by continuation maintenance therapy. Continuation maintenance therapy did not advance the line of therapy and was defined as the continued administration of pemetrexed, erlotinib, or bevacizumab after their use (one or more) in combination with other drugs when the other drugs were dropped (see online supplement for further details). Switch maintenance was not discernable in this study because disease progression information was unavailable, with treatment switching always representing a new line of therapy.

OS was defined as the time period from index date until death. If no date of death was observed, the survival time was censored at the date of last follow-up. All death dates were generalized to the 15th of the month for de-identification reasons. OS analyses were conducted for the overall cohort, and by histology. While the requirement of two visits on/after 2013 was necessary to select a cohort of patients relevant for the examination of contemporary trends in the treatment of advanced NSCLC, this criterion may have introduced a survival bias. To account for the potential survival bias introduced by the visit criteria, a sensitivity analysis was conducted on the subgroup of patients with an advanced diagnosis date on/after 2013.

### Statistical analysis

Patient characteristics and treatment patterns, both overall and by tumor histology (squamous vs. nonsquamous), were described using summary statistics, including frequencies and percentages for categorical variables and mean and standard deviation (SD), median and interquartile range (IQR), and range for continuous or interval data, as appropriate. Survival curves for OS were plotted using the Kaplan-Meier method. Medians, and the associated 95% confidence intervals, were computed.

We did not conduct a formal calculation of sample size and statistical power because this was a descriptive study without hypothesis testing. Statistical analyses were performed using SAS 9.3 software (SAS Institute Inc., Cary, NC).

## Results

### Patient characteristics

**Fig 1:**
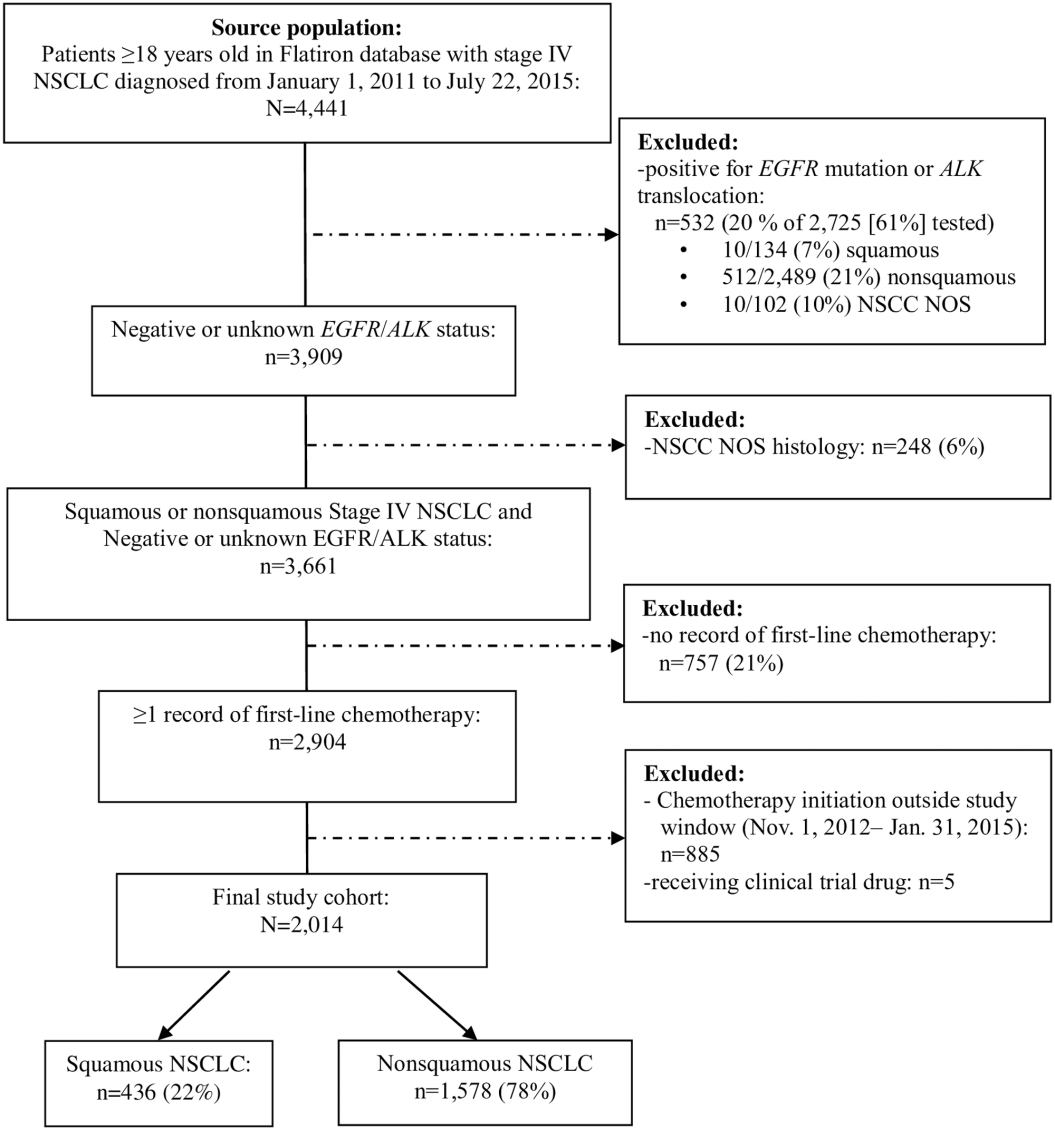
Patient flow chart. Abbreviations: NSCLC, non-small cell lung cancer; NSCC NOS, non-small cell carcinoma, not otherwise specified.

The source population for this study included 4,441 patients ≥18 years of age with stage IV NSCLC diagnosed between January 1, 2011, and July 22, 2015, identified in the Flatiron database (see patient flow chart, Fig 1). Of the 3,661 patients with stage IV NSCLC without known *EGFR* or *ALK* genomic tumor aberrations or NSCLC NOS histology, 2,904 (79%) patients had at least one record of first-line chemotherapy. The final study population comprised the 2,014 patients who had documented first-line chemotherapy initiated from November 1, 2012, through January 31, 2015, including 436 (22%) with squamous and 1,578 (78%) with nonsquamous NSCLC. Baseline patient characteristics are summarized in Table 1 for the full study population and by histology; mean (SD) age was 67 (10) years, 55% were male, and 87% had a history of smoking. Patients with squamous NSCLC were more commonly male (64% vs. 53%), older (34% vs. 25% of age ≥75 years) and smokers (90% vs. 86%) than those with nonsquamous histology. The number of patients not tested for EGFR mutation was 368 (84.4%) for squamous, 460 (29.2%) for non-squamous, and 828 (41.1%) overall. The number not tested for ALK translocation was 368 (84.4%) for squamous, 487 (30.9%) for non-squamous, and 855 (42.5%) overall. The most common methods of detecting EGFR mutation was polymerase chain reaction (PCR) (54.4%), unknown (23.2%), other (14%), and next generation sequencing test (NGS) (8.4%). 76.4% of the ALK mutations were detected using a fluorescence in situ hybridization (FISH) test, followed by 13.2% using a other test, 5.6% using an NGS test and 0.9% using an immunohistochemistry (IHC) test.

**Table 1:** Characteristics of patients presenting with stage IV NSCLC.

	Squamous	Nonsquamous	All Patients
Characteristics	n = 436	n = 1,578	N = 2,014
Sex, male	280 (64.2)	829 (52.5)	1,109 (55.1)
Age at index date, mean (SD)	69.6 (9.3)	66.8 (10.2)	67.4 (10.1)
Range	31–84	28–85	28–85
Age at index date			
<45	4 (0.9)	30 (1.9)	34 (1.7)
45–54	22 (5.1)	150 (9.5)	172 (8.5)
55–64	92 (21.1)	444 (28.1)	536 (26.6)
65–74	170 (39.0)	555 (35.2)	725 (36.0)
≥75	148 (33.9)	399 (25.3)	547 (27.2)
Race			
Caucasian	280 (64.2)	980 (62.1)	1,260 (62.6)
Black	32 (7.3)	135 (8.6)	167 (8.3)
Asian	4 (0.9)	33 (2.1)	37 (1.8)
Unknown	120 (27.5)	430 (27.3)	550 (27.3)
History of smoking*	394 (90.4)	1,352 (85.7)	1,746 (86.7)
BMI at index date, mean (SD)†	26.1 (5.9)	26.0 (5.5)	26.0 (5.6)
Range	14–53	12–51	12–53
Geographic region‡			
Midwest	99 (22.7)	342 (21.7)	441 (21.9)
Northeast	98 (22.5)	375 (23.8)	473 (23.5)
South	175 (40.1)	585 (37.1)	760 (37.7)
Unknown	5 (1.2)	24 (1.5)	29 (1.4)
West	59 (13.5)	252 (16.0)	311 (15.4)
EGFR			
Negative	67 (15.4)	1,049 (66.5)	1,116 (55.4)
Unknown	1 (0.2)	69 (4.4)	70 (3.5)
Not tested	368 (84.4)	460 (29.2)	828 (41.1)
ALK			
Negative	65 (14.9)	1,002 (63.5)	1,067 (53.0)
Unknown	3 (0.7)	89 (5.6)	92 (4.6)
Not tested	368 (84.4)	487 (30.9)	855 (42.5)
First-line induction therapy only	433 (99.3)	1246 (79.0)	1679 (83.4)
First-line induction followed by continuation maintenance therapy	3 (0.7)	332 (21.0)	335 (16.6)
Received second-line therapy	169 (38.8)	670 (42.5)	839 (41.7)
Received third-line therapy	92 (21.1)	346 (21.9)	438 (21.7)

Note: Data are n (%) unless otherwise indicated.

Abbreviations: BMI, body mass index; NSCLC, non-small cell lung cancer.

*Smoking history data were missing for 17 (4%) and 56 (4%) patients in squamous and nonsquamous cohorts, respectively.

^†^BMI data were missing for 11 (3%) and 43 (3%) patients in squamous and nonsquamous cohorts, respectively.

^‡^Geographic region data were missing for 29 (1.4%) overall.

### First-line chemotherapy

Platinum-based combination regimens were the most commonly used first-line chemotherapy overall (1714; 85%), including for both patients with squamous (380; 87%) and nonsquamous histology (1334; 85%) (Table 2). Carboplatin doublet regimens were prescribed to approximately half of patients overall (1,077; 53%), including 333 (76%) and 744 (47%) patients in squamous and nonsquamous NSCLC cohorts, respectively. Carboplatin doublet plus bevacizumab were prescribed to 469 (23%) patients overall, including 1% and 29% in squamous and nonsquamous cohorts, respectively. Cisplatin doublet regimens were prescribed less frequently: 88 (4%) patients overall, including 4–5% in each cohort.

**Table 2:** Induction regimens administered to 1% or more of patients in either squamous or nonsquamous cohorts, by histology and overall.

Induction regimen*	Squamous (n = 436)	Nonsquamous (n = 1,578)	All Patients (N = 2,014)
Carboplatin+pemetrexed	3 (0.7)	405 (25.7)	408 (20.3)
Carboplatin+paclitaxel	164 (37.6)	220 (13.9)	384 (19.1)
Carboplatin+bevacizumab+pemetrexed	0	252 (16.0)	252 (12.5)
Carboplatin+bevacizumab+paclitaxel	4 (0.9)	171 (10.8)	175 (8.7)
Carboplatin+ nab-paclitaxel	92 (21.1)	37 (2.3)	129 (6.4)
Erlotinib	13 (3.0)	58 (3.7)	71 (3.5)
Carboplatin+gemcitabine	51 (11.7)	16 (1.0)	67 (3.3)
Pemetrexed	2 (0.5)	64 (4.1)	66 (3.3)
Carboplatin+docetaxel	21 (4.8)	38 (2.4)	59 (2.9)
Cisplatin+pemetrexed	0	44 (2.8)	44 (2.2)
Docetaxel	6 (1.4)	24 (1.5)	30 (1.5)
Gemcitabine	8 (1.8)	21 (1.3)	29 (1.4)
Carboplatin+etoposide	1 (0.2)	25 (1.6)	26 (1.3)
Cisplatin+etoposide	6 (1.4)	15 (1.0)	21 (1.0)
Carboplatin+bevacizumab+docetaxel	0	16 (1.0)	16 (0.8)
Bevacizumab+pemetrexed	0	16 (1.0)	16 (0.8)
Cisplatin+gemcitabine	11 (2.5)	6 (0.4)	17 (0.8)
Carboplatin+paclitaxel/nab-paclitaxel	10 (2.3)	6 (0.4)	16 (0.8)
Vinorelbine	7 (1.6)	8 (0.5)	15 (0.7)
Paclitaxel	6 (1.4)	11 (0.7)	17 (0.8)
nab-Paclitaxel	5 (1.2)	5 (0.3)	10 (0.5)
Other regimens†	26 (6.0)	120 (7.6)	146 (7.2)

Data are n (%).

Abbreviation: nab-paclitaxel, albumin-bound paclitaxel particles

*Induction regimen includes patients who received induction therapy only or patients who received induction followed by continuation maintenance.

^†^”Other” regimens were those received by <1% of patients in each cohort (squamous or nonsquamous)

Overall, regimens prescribed to >10% of all patients were carboplatin plus pemetrexed (20%), carboplatin plus paclitaxel (19%), and carboplatin, pemetrexed, and bevacizumab (13%). These three regimens were predominant for the nonsquamous cohort as well (26%, 14%, and 16%, respectively), and regimens prescribed to >10% of patients in the squamous cohort were carboplatin plus paclitaxel (38%), carboplatin plus albumin-bound paclitaxel particles (nab-paclitaxel; 21%), and carboplatin plus gemcitabine (12%) (Table 2). In the nonsquamous cohort, the overall regimen duration range was 1–566 days; median regimen durations were 57–92 days (except for erlotinib). In the squamous cohort, the overall range was 1–422 days, with longest median regimen duration with carboplatin plus nab-paclitaxel (120 days), followed by carboplatin plus gemcitabine (92 days).

Of the 2,014 patients who received first-line chemotherapy, 839 (42%) continued to a second-line regimen, and 438 (52%, or 22% overall) continued to a third-line regimen (Table 1) during the study follow up period. Fifty-one (2.5%) patients received a PD-1 inhibitor as second or later line of therapy.

### Continuation maintenance therapy

A total of 332 nonsquamous patients received continuation maintenance therapy (26.6%; 332/1246). Continuation maintenance regimens were divided among pemetrexed alone (n = 142/332; 42.8%), bevacizumab alone (n = 86/332; 25.9%), pemetrexed plus bevacizumab (n = 73/332; 22.0%), and other (n = 31/332; 9.3%). Three squamous patients (3/346; 0.7%) received continuation maintenance.

### Overall survival

**Fig 2:**
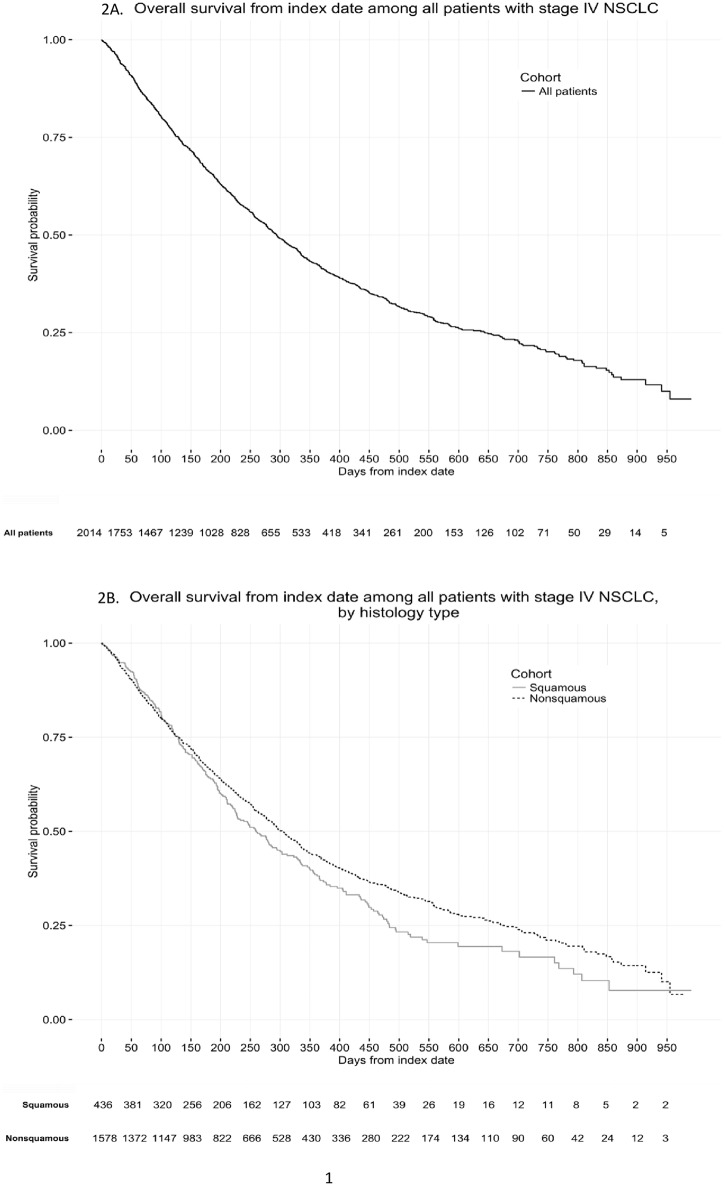
(A) Kaplan-Meier plot of overall survival analyses among patients with stage IV NSCLC. Note: The median was 294.0 days (95% CI: 277.0, 314.0). (B) Kaplan-Meier plot of overall survival analyses among patients with stage IV NSCLC, by squamous and nonsquamous histology. Note: The median OS was 260.0 days (95% CI: 226, 303.0) for squamous and 304.0 (95% CI: 286.0, 329.0) for nonsquamous NSCLC.

Median OS for the overall cohort (n = 2014) is shown in Fig 2A, where median OS was 294 days (9.7 months) (95% CI: 277, 314). The OS Kaplan-Meier plot for patients by histology is shown in Fig 2B. The OS curves are indistinguishable for squamous and nonsquamous sub-groups during the first 120 days, but higher survival probabilities were observed for nonsquamous than squamous NSCLC after this time point. The median OS was 260 days (8.5 months) (95% CI: 226, 303 days) for squamous and 304 (10.0 months) (95% CI: 286, 329 days) for nonsquamous NSCLC. In sensitivity analyses, survival was similar when restricting the inclusion criteria to patients diagnosed on or after January 1, 2013, with a median OS of 292 days for the overall cohort, 256 days for the squamous cohort, and 300 days for the nonsquamous cohort.

## Discussion

This study responds to repeated requests from clinicians, regulators and payers: What is the real-world impact of the most commonly prescribed treatments? New cancer treatments are rapidly being approved, and several are advancing into the first line setting; the overall impact of the introductions of these drugs needs to be compared against a contemporary real-world baseline. Here we focus on real-world treatment patterns and OS outcomes for people diagnosed with stage IV NSCLC without known *EGFR* or *ALK* tumor aberrations in the US community oncology setting during a contemporary time period just before immune checkpoint inhibitors entered standard of care in the US. This study sets an important baseline as we start to evaluate, as a whole, whether ongoing shifting treatment patterns in the US are making a difference on patient outcomes.

Overall, 79% of the 3,661 patients in this representative cohort initiated systemic anticancer therapy, 22% with squamous and 78% with nonsquamous histology. Platinum-based combination regimens were the most common first-line therapy (85%), usually administered as a carboplatin doublet regimen with (23%) or without (53%) bevacizumab therapy. The majority of the different regimens prescribed for first-line induction and continuation maintenance accorded with treatment guidelines [19]. Median OS was 9.7 months for the study population, and 8.5 and 10.0 months for squamous and nonsquamous patients respectively.

Only several published studies describe real-world practice patterns of first-line therapy for NSCLC in the US [10,11,20–22]. The two most recent studies—Zornosa et al [10] (2006–2009 data) and Henk et al [10] (2006–2010 data)—were from periods before approval of maintenance therapies [23, 24] and identification of the importance of histology in chemotherapy selection [25]. Our study, confirms several prior observations. First, the proportion of metastatic NSCLC patients receiving first line therapy has risen over time from 52% in 2000 [22] and 59% in 2007, [22] to 79%-80% in 2009 [10] and here. Second, consistent with guidelines, a carboplatin doublet is the most common induction regimen [4,5,10,20,21], followed by carboplatin doublet and bevacizumab [10,11,21].

The current study extends our understanding of contemporary treatment patterns and outcomes by documenting OS results in patients without actionable mutations, stratification of results by squamous and nonsquamous histology, and the reporting of both induction and continuation maintenance regimens, by regimen type and histology. Proportions of patients with squamous vs. nonsquamous histology (22% vs. 78%) and their relative characteristics (more likely male, older, and smoker in the squamous cohort) were consistent with NSCLC population estimates [1]. In the squamous cohort, the three most commonly used induction regimens were carboplatin plus paclitaxel (38%), carboplatin plus nab-paclitaxel (21%), and carboplatin plus gemcitabine (12%). In the nonsquamous cohort, carboplatin plus pemetrexed was the dominant regimen, used either with (16%) or without bevacizumab (26%). Carboplatin plus paclitaxel was the next most commonly used regimen either with (11%) or without bevacizumab (14%). Continuation maintenance was used in a minority of nonsquamous patients (26%).

This is the first observational study, to our knowledge, of OS in first line metastatic stage IV NSCLC patients without known actionable mutations. We observed a slightly higher median OS among our squamous patients (8.5 months) compared to Davis et al [20] reporting median OS of 8 months in a large cohort of Medicare enrollees with metastatic squamous NSCLC. The difference in median survival time could be explained partly due to large number of older enrollees in Medicare cohort compared to our real-world community oncology population (mean [SD] age 74.6 [5.9] versus 67.4 [10.1] years). The median OS from this study was comparable with a previous study reporting median survival in stage IV NSCLC (8.7 (6–9.8) months) irrespective of EGFR or ALK status, illustrating minimal improvement in OS and a huge unmet need in this patient population [26]. A European study reported a median OS was 10.3 mos. (95% CI: 7.4–11.8) months which was and consistent with our study results for patients receiving platinum based chemotherapy in first line treatment between 2009 and 2011[27]. A population-based study in Ontario, Canada study reported OS for 1511 metastatic NSCLC diagnosed between 2005–2009 receiving the most common treatments, with median OS of 11.6 months for cisplatin plus gemcitabine and 8.4–9.3 months for other regimens, which was also consistent with our results.[28]

In addition to the contemporary findings, other strengths of this study include the description of first-line continuation maintenance treatment. By using a nationally representative dataset including both structured plus unstructured data processing, results are generalizable to the US community oncology setting [16]. Moreover, the large patient population was drawn from community oncology practices throughout different regions of the US, providing an important snapshot of the majority of care received in the US. The study design enabled a minimum of 6-months’ follow-up for each patient, while also ensuring that OS outcomes are representative of the period just before immune checkpoint inhibitors became commonplace in the US.

## Limitations

A limitation of our findings is the potential for misclassification of continuation maintenance therapy using database algorithms. The algorithm used in this study did not capture switch maintenance therapy, which is difficult to capture because two of the three maintenance therapy drugs (erlotinib and pemetrexed) have FDA indications for second-line use as monotherapy [29,30]. For the purposes of this study, continuation maintenance therapy was limited to pemetrexed, bevacizumab, and erlotinib. Also, some clinically important patient characteristics were not available in the database (e.g., performance status, detailed smoking history, comorbidities), additional treatment modalities (e.g., surgery, radiation therapy), and clinical outcomes (e.g., disease progression, mortality); moreover, *EGFR/ALK* status was available only for those who were tested. In addition, we could not be certain whether oral drugs (e.g., erlotinib) were taken as prescribed. The dates of death were based only on the month of death, due to data de-identification reasons—all death dates were assumed to occur on the 15^th^ of the month. Finally, the data are drawn from community oncology centers and thus may not be representative of treatment patterns at academic medical centers.

## Conclusions

This study provides the first contemporary real-world snapshot of treatment patterns and outcomes for stage IV NSCLC patients without targetable mutations in the US community oncology setting. It presents a baseline for OS in this population, and documentation of true unmet medical need for this group of individuals at risk of shortened lives due to their disease. As new treatments enter the therapeutic landscape, our combined task as clinicians, researchers, manufacturers, payers, regulators and society is to advance treatments that will meaningfully move this needle. Also, we must get progressively more sophisticated in patient selection for optimal therapies through biomarkers, assessment of patient fitness, determination of best treatment sequencing and demonstration of what works for whom and when. By describing OS for stage IV NSCLC, the results of this study provide a necessary starting point for benchmarking high-quality care as new therapies for NSCLC are introduced into clinical practice.

## Supporting information

S1 File Online supplement. (PDF)
