# Supplementary material for: Real-world first-line treatment and overall survival in non-small cell lung cancer without known EGFR mutations or ALK rearrangements in US community oncology setting
Source: PLoS One. 2017 Jun 23;12(6):e0178420. doi: 10.1371/journal.pone.0178420 (PMC5482433; doi:10.1371/journal.pone.0178420)
Supplement: S1 File — (PDF) [file pone.0178420.s001.pdf]

Online Supplement

Real-world First-Line Treatment and Overall Survival in Non-Small Cell Lung Cancer without Actionable Mutations

Abernethy A, et al.

Supplemental Methods

Comorbidities were identified from the electronic health record (EHR) diagnosis workflow. Records were excluded if the diagnosis could not be mapped to a valid International Classification of Diseases, Ninth Revision, Clinical Modification (ICD-9-CM) diagnosis code.[1] Disease characteristics, specifically diagnosis date and recurrence date, were determined by abstraction of unstructured data available in the EHR. Unstructured data processing took place using Flatiron Health's technology-enabled abstraction approach, as outlined in the parent IRB protocol. A metastatic recurrence was determined by comparing the diagnosis_date and advanced_diagnosis_date variables: patients with an advanced_diagnosis_date after the diagnosis_date were considered as having metastatic recurrence.

Lines of Therapy Rules

The start of a line of therapy was defined as the first drug episode (drug order or administration) of a specified list of medications, plus any other drug episodes (including new drugs) that occurred within 28 days of the first episode. Any subsequent drug episodes of the drugs received in the first 28 days were included as part of the first line of therapy and did not advance the line. A gap of >120 days between administrations of the same drug advanced the line of therapy. If a patient received a new combination of drugs from the pre-specified list, the line of therapy did

not advance in the following cases: substitution of cisplatin for carboplatin or vice versa, substitution of paclitaxel for albumin-bound paclitaxel particles (nab-paclitaxel), or addition of bevacizumab to a chemotherapy or targeted therapy background.

Treatment duration was defined as the time from regimen initiation to the last dose of drugs within the regimen and was summarized for patients receiving only first-line therapy as well as those receiving first-line (induction) followed by maintenance therapy. Patients who initiated the next line of therapy or who had at least 30 days without any treatment after the last dose of the first-line regimen were assumed to have discontinued the first-line regimen. Treatment duration was described by histology for each regimen category, as well as for the most commonly used regimens.

Maintenance therapy did not advance the line of therapy and was defined as the continued administration of pemetrexed, erlotinib, or bevacizumab after their use (one or more) in combination with other drugs when the other drugs were dropped. A switch to erlotinib or pemetrexed monotherapy advanced the line of therapy and was not considered as maintenance. Similarly, the addition of erlotinib or pemetrexed to a chemotherapy or targeted therapy regimen advanced the line of therapy, while the addition of bevacizumab to a chemotherapy or targeted therapy regimen did not advance the line of therapy. However, if bevacizumab was continued when one or more of the background agents were dropped, that was considered as maintenance. Information about gemcitabine prescribing for maintenance therapy was not designated in the database.

Reference

1. International Classification of Diseases, Ninth Revision, Clinical Modification (ICD-9-CM). http://www.cdc.gov/nchs/icd/icd9cm.htm
